# Evaluation of balance in fallers and nonfallers elderly

**DOI:** 10.5935/1808-8694.20120016

**Published:** 2015-11-20

**Authors:** Banu Müjdeci, Songul Aksoy, Ahmet Atas

**Affiliations:** a Evaluation of Balance in Fallers and Non-Fallers Elderly (Numune Training and Research Hospital, Center of Hearing, Speech and Vestibular Disorders Diagnosis and Treatment, Ankara, Turkey, MS); b Assoc. Prof (Division of Audiology and Speech Pathology, Department of Ear Nose and Throat Faculty of Medicine, University of Hacettepe, Ankara, Turkey, Assoc. Prof.); c Assoc. Prof (Cerrahpasa Faculty of Medicine, Department of Audiology, University of Istanbul, Istanbul, Turkey, Assoc. Prof.)

**Keywords:** accidental falls, elderly, medicare, postural balance

## Abstract

Falls present a substantial health problem among the elderly population. Approximately one-third of community-dwelling people over 65 years of age will experience one or more each year.

**Objective:**

The purpose of this study was to evaluate balance between fallers and non-fallers elderly. Study Design: Clinical study.

**Methods:**

We studied 30 subjects older than 65 years of age. 15 subjects had a history of falls within a year (Group I) and 15 subjects had no history of falls (Group II). The scores of Computerized Dynamic Posturography (CDP); Sensory Organization Test (SOT), Limits of Stability (LOS), Rhytmic Weight Shift (RWS) and Berg Balance Scale (BBS) findings gathered from the individuals from Group I and Group II, were compared.

**Results:**

The SOT 3, 6, composite, BBS scores and left-right on-axis velocity score of RWS test of the Group I were found to be significantly lower the Group II (*p* < 0.05). A positive correlation between the SOT 3, 5, composite and BBS scores of Group I and the SOT 4, 5, 6, composite and BBS scores of Group II is determined (*p* < 0.05).

**Conclusion:**

The CDP and BBS scores in fallers were found to be significiantly lower as compared to the non-fallers elderly.

## INTRODUCTION

Falls present a substantial health problem among the elderly population. Approximately one-third of community-dwelling people over 65 years of age will experience one or more each year^1^. To maintain postural stability while standing and walking, the brain must rapidly process signals from the visual, vestibular, and somatosensory systems. Because balance depends on multiple sensory inputs it can deteriorate when any of these systems fails individually or collectively. The combined loss of sensory signals from several systems has been proposed as a common cause of imbalance (so-called multisensory dizziness and imbalance)^2^.

Deterioration in balance function, whether a natural process related to aging or as a result of disease, is observed much more often in the elderly population than it is within younger individuals^3^. A recent 25 year study has shown that not only has the number of falls in the elderly population increased but also the incidence of fall- induced injuries and deaths has increased significantly^4^.

Nearly 40% of falls occuring in the 65 years of age and over population are admitted to the hospital for some type of treatment^1^. In 5% of the cases, the injuries suffered include fractures, bruises, soft tissue injuries and loss of self-confidence^5^. As a result, the elderly population develops a fear of falling complex, a decrease in self confidence to accomplish normal activities of daily living and adopt a lifestyle of inactivity resulting in significant muscular atrophy, most noticable in lower extremity strength^6^.

A simple predictive models were reported using logistic regressions that combined Berg Balance Scale (BBS) scores with a self reported history of imbalance to predict risk of falls^1^. Some authors proposed that balance (postural stability) requires three distinct processes: (i) sensory organization, in which one or more of the orientational senses (somatosensory, visual and vestibular) are involved and integrated within the central nervous system; (ii) a motor adjustment process involved with executing coordinated and properly scaled neuromuscular responses; and (iii) the background tone of the muscles, through which changes in balance are effected^7^.

Posturography, which is a measurement of body sway, may be a useful technique for quantifying imbalance in older persons and identfying those at risk for falling. Several researchers have shown that sway incrases in older persons^8^, and the research has linked greater amounts of postural sway to increased risk of falling, which is a serious problem for older adults^9^. Researchers found significiantly greater antero-posterior sway in those older adults who had a fall in the 1-year period following the balance measurement. Early detection of abnormalities in dynamic postural control followed by appropriate rehabilitation, environment modification and recommendations may help prevent falls, thereby substantially improving the quality of life of elderly individuals^9^.

Measurements obtained with the BBS show high specifity but poor sensitivity, for identifying people with increased risk of falling. The BBS, however, shows sensitivity and specificity to predict use of assistive devices in the older adults. The BBS is easy to administer and requires no special equipment^10^.

This study was designed to identify balance impairments associated with falling in elderly subjects. The purpose of this study was to evaluate balance between fallers and non-fallers amongst the elderly.

## METHOD

We studied 30 subjects older than 65 years of age. Fifteen of the subjects (mean age of 70.20 ± 4.39 years) having a history of at least two spontaneous falls within a year with no loss of consciousness or detectable cause (e.g. sudden paralysis, seizure or heavy drinking) constituted Group I, the other 15 subjects (mean age of 71.93 ± 6.11 years) having no history of falls constituted Group II.

The study was approved by the Ethical Committee of the institution under number LUT08/11. After the scope and objective of the research had been explained to the individuals who participated in the research and their relatives, their written consents were also obtained.

Inclusion criteria required for the elderly subjects: no histories of significant head trauma, neurological disease (e.g Parkinson's, post-polio syndrome, diabetic neuropathy), visual impairment not correctable with lenses, musculoskeletal impairments (e.g. amputation, joint replacement, joint fusions, joint deformity due to rheumatoid arthritis), or persistent symptoms of vertigo, light-headedness, unsteadiness. A fall was defined as any event in which the individual lost their balance and made contact with the floor (i.e did not simply fall back into a chair after trying to stand up).

All subjects were evaluated in a study of postural stability and balance using the BBS and Computerized Dynamic Posturography (CDP). The Sensory Organization Test (SOT) 1, 2, 3, 4, 5 and 6, besides the Limits of Stability Test (LOS) and Rhytmic Weight Shift Test (RWS) are subtests of the CDP. The SOT, LOS and RWS test protocols were administered using the Smart Balance Master (Neurocom International, Inc., Clackamas, OR, USA).

The SOT was performed in a clinically routine manner. SOT included six tests conditions:

• SOT 1. Eyes open, fixed support surface and surround (visual, vestibular, and somatosensory modalities available);
• SOT 2. Eyes closed, fixed support surface and surround (absent visual input);
• SOT 3. Eyes open, sway-referenced surround, and fixed support surface (visual input inaccurate);
• SOT 4. Eyes open, sway-referenced support surface, and fixed surround (somatosensory inputs inaccurate);
• SOT 5. Eyes closed, sway-referenced support surface, and fixed surround (absent visual input and somatosensory input inaccurate);
• SOT 6. Eyes open, sway-referenced surround and support surface (inaccurate visual and somatosensory inputs).

The LOS testing provides information on the patient's skills in moving the center of gravity over the base of support while maintaining an upright posture as individuals are asked to sway, using ankle strategies and weight shifts only, forward, backward, to the left and to the right. Test measures included maximum endpoint excursion for anterior, posterior, right, and left movements and were measured as a percentage of the maximum end point reached during an 8-second trial.

The RWS assessment quantifies two movement characteristics associated with the patient's ability to voluntarily move their center of gravity or “sway” from left-to-right and forward-to-backward in a rhythmic manner. The measured parameters are on-axis velocity and directional control.

BBS, consist of 14 subtests performed in a standard order. Each task scored on a five-point scale (0-4) according to quality of the performance or the amount of time needed to complete the task, as ranked by the test developers. The maximum score for this assessment is 56.

We classified the subjects based on their falls history as a Group I (fallers) and Group II (non-fallers). BBS, SOT, RWS, LOS scores of groups were compared using the independent *t* test.

We analyzed the relationship between linear related variables using Pearson correlation analyses.

Study data were analyzed using the SPSS statistical package (version 15). Statistical significance was determined at *p* less than 0.05 for all analyses.

## RESULTS

As two groups were compared, the SOT 3, 6, composite scores of the Group I was found to be statistically significantly lower from the Group II (*p* < 0.05) (Table 1). There was no statistically significantly difference detected between two groups regarding the SOT 1, 2, 4, 5 scores (*p* > 0.05).

**Table 1:** SOT figures of the individuals.

SOT	Group I (Mean ± SD)	Group II (Mean ± SD)	*P*
SOT 1	91.93 ± 3.35	93.53 ± 1.57	NS
SOT 2	90.96 ± 4.16	93.10 ± 2.63	NS
SOT 3	88.73 ± 3.54	92.96 ± 2.15	.000*
SOT 4	76.86 ± 6.99	78.30 ± 8.23	NS
SOT 5	66.46 ± 9.74	72.86 ± 7.28	NS
SOT 6	60.40 ± 11.14	71.10 ± 6.55	.003*
Composite	77.06 ± 3.47	81.66 ± 3.37	.001*

SD: Standart deviation

NS: Not significant (*p* > 0.05).

* Independent *t*test (*p* < 0.05 was significant)

In this study, the BBS score average of the Group I was found to be 47.9 and of the Group II to be 54.6. As two groups were compared to each other, the BBS score of the Group II was found to be statistically significantly higher than the Group I (*p* < 0.05).

The on-axis velocity score during left/right movement of the RWS test was found to be statistically significantly low in the Group I than the score of the Group II (*p* < 0.05).

There was no statistically significant difference detected between two groups regarding the left/right directional control score, forward/backward directional control score and on-axis velocity score during forward/backward movement of RWS and LOS scores (*p* > 0.05).

As the correlations between BBS, SOT, LOS and RWS scores were evaluated separately within the two groups, a positive correlation between the SOT 3, 5, composite scores and BBS score was detected within the Group I (*p* < 0.05).

Positive correlation between the SOT 4, 5, 6, composite scores and BBS score within the Group II was also detected (*p* < 0.05).

## DISCUSSION

This study reports comparative results of CDP and BBS tests carried out on either group amoung which Group I consist of 15 elderly subjects who are reported have experienced two or more unexpected falls during the past 12 months and Group II which includes elderly people that are non-fallers (n = 15).

CDP has become an important tool for comprehending standing balance in clinical settings. A key test in the Neurocom International (Clackamas, Oregon) Dynamic Posturography System, the SOT, provides information about the integration of multiple components of balance. The SOT test leads to an outcome measure called the “equilibrium score”, which reflects the overall coordination of the visual, proprioceptive, and vestibular systems for maintaining standing posture^11^.

Some authors found that discriminant function analysis identified visual contrast sensitivity, lower limb proprioception, quadriceps strength, reaction time and sway on foam with the eyes open as the variables that significantly discriminated between subjects who experienced multiple falls and subjects who experienced one fall or fewer^12^.

In the study found that effects of ageing are loss of cutaneous sensation, which appears to correlate with impaired postural control and an increased risk of falling^13^.

Some researcher evaluated 100 elderly individuals in order to analyse the correlation between falling and SOT scores within individuals who suffer from balance disorders. As a result, they determined that the SOT scores within the individuals who are recurrent fallers were significantly lower when compared to the individuals who were one-time fallers. They stated that the CDP performance within the planning the safe exercise program can be helpful to the clinician^14^.

In the study the SOT scores within the faller and non-faller individuals aged 60 and over were compared and a statistically significant difference between two groups regarding the composite score was detected. The author stated that SOT tests could be used in order to determine the difference in terms of balance disorders within faller and non-faller elderlies^15^.

In our study, the SOT 3, 6 and composite scores of the individuals in the Group I was found to be statistically significantly lower as compared to those of the individuals in the Group II (*p* < 0.05).

This conclusion approved the results of the studies that detected a significant correlation between the SOT and falling^14^^,^^15^. The finding we concluded in our study indicates that the dynamic balance within the elderly people who are fallers was negatively affected. We strongly believe that SOT will be very useful in determining the risk of falling among elderly people.

The study compared the electronystagmography and CDP in determining the risk of falling within 33 individuals and concluded that the CDP and especially LOS tests provide important information regarding the risk of falling within elderly people^16^. In their studies (n = 273), researchers stated that LOS provides information on postural deficits with the people who have potential to fall^17^.

Some researchers stated in their study that was conducted on 19 individuals reported to have fallen and on 124 non-faller individuals (average age is 78) in which they utilized LOS in order to analyse the postural stability, that LOS was a very beneficial clinical practice in forecasting the risk of falling. There was no significant difference determined between the two groups regarding the LOS scores^18^. This finding did not verify the study^18^ that states LOS can be used for forecasting the risk of falling.

Some authors, evaluated the dynamic balance within 202 individuals aging 60 and over fallers (n = 59) and non-fallers (n = 143). They determined failures within the postural controls and more failures within the lateral balance on dynamic postural controls with fallers as they were compared to non-fallers. They detected control of lateral stability was the most severely impaired component of postural control in the fallers^19^.

In this study the on-axis velocity score during left/right movement of RWS in the Group I was found to be statistically significantly lower as compared with the individuals in the Group II (*p* < 0.05). This conclusion verified the literature research^19^ that concludes the lateral balance with the individuals who are fallers was negatively affected. There was no statistically significant difference detected between two groups regarding the left/right directional control score, forward/backward directional control and on-axis velocity score during forward/backward movement (*p* > 0.05).

The BBS was easy to administered and required no special equipment. We believed that the determination of risk of falling within patients, might be substantially improved by also examining their environment and how well they complete their daily activities. BBS showed sensitivity and specificity to predict use of assistive devices within the older adults^10^.

In the study analyzing the factors that contribute to falling within elderly people (n = 125), it is found that the BBS score of the fallers are significantly lower when compared to non-fallers^20^. Another study determined that BBS score average of fallers was 39.6 whereas 52.6 with the non-fallers^1^.

Some authors concluded that the average BBS score of fallers was 36 and of non-fallers was 50.4^20^.

In this study the average BBS score of the individuals in the Group I and Group II were found to be 47.9 and 54.6 respectively. As the two groups were compared, the BBS score of the individuals in the Group II was found to be statistically significantly higher than the BBS score of the Group I (*p* < 0.05). This result means that the body balances of the faller individuals, are negatively affected. This finding matches up with the literature researches^1^^,^^20^ concluding the BBS was the determiner of the risk of falling within the elderly people. Moreover, in our study we evaluated the relation between composite score and BBS within both of the groups.

We detected that there were positive correlations between SOT 3, 5, composite scores and BBS score in the Group I and SOT 4, 5, 6, composite scores and BBS score in the Group II (*p* < 0.05). These results indicated that scores relating to dynamic balance in SOT are positively correlated to BBS scores within both groups.

In our study, considering the positive correlation obtained between the BBS score and scores relating to dynamic balance within SOT in both groups, SOT can be used for the exact same purposes as the BBS (*p* < 0.05). The clinics who are not able to afford complicated devices like CDP, can use BBS for evaluating balance.

In this study, finding significantly lower SOT parameters that evaluate the dynamic balance and BBS score in the fallers group; indicates that especially dynamic balance is negatively affected within the elderly people who are fallers. With the help of using efficient therapy strategies for elderly people who has the risk of falling, the falls and serious injuries that are caused by falls can be prevented.

## CONCLUSION

Our results showed that the CDP and BBS scores in fallers were found to be significiantly lower as compared to the non-fallers. These measurements can be used for determining the balance impairments associated with falling in elderly.
